# Analysis of mitochondrial DNA variations in Indian patients with congenital cataract

**Published:** 2012-01-24

**Authors:** Mascarenhas Roshan, Shama Prasada Kabekkodu, Pai H. Vijaya, Kamath Manjunath, Jochen Graw, PM. Gopinath, Kapeattu Satyamoorthy

**Affiliations:** 1 Division of Biotechnology, Manipal Life Sciences Centre, Manipal University, Manipal, India; 2 Department of Ophthalmology, Kasturba Hospital, Manipal University, Manipal, India; 3 Department of Ophthalmology, Government Wenlock Hospital, Mangalore, India; 4 Helmholtz Center Munich, German Research Center for Environmental Health, Institute of Developmental Genetics, Neuherberg, Germany

## Abstract

**Purpose:**

Identification of mitochondrial DNA (mtDNA) variations in the inherited cataract patients from south India.

**Methods:**

Three families with inherited cataract of maternal origin were evaluated. Clinical and ophthalmologic examinations were performed on available affected as well as unaffected family members. Samples of mtDNA were amplified using 24 pairs of overlapping primers to analyze the entire mitochondrial genome to screen for variations and analyzed for both coding and non-coding regions. Bioinformatic analysis was performed to evaluate the effect of nucleotide variations.

**Results:**

DNA sequence analysis of inherited cataract families showed 72 nucleotide variations, of which 15 were observed in the major non-coding D-loop region, 3 in the tRNA genes, 5 in the rRNA genes, and 49 in the protein coding region. Among these variations 56 were reported previously and 16 were novel of which, 12 synonymous substitutions, 2 non-synonymous substitutions along with a frameshift mutation, and one was in the non-coding region. Nicotinamide adenine dinucleotide dehydrogenase (NADH) subunit (*ND*) gene of mtDNA was highly altered, in general, and found to contain 4 variations specific for cataract patients of the first family, six for the second, and one for the third family.

**Conclusions:**

Seventy-two variations were observed in three inherited cataract families. Four variations were specific for cataract patients of the first family, six for the second, and one for the third family. This is perhaps the first report on the presence of mitochondrial mutations in inherited cataracts.

## Introduction

Cataracts are the leading cause of blindness in children with an estimated incidence of 1–6 per 10,000 live births. About 8.3%–25% of cataracts are familial and inherited in a Mendelian fashion predominantly as autosomal dominant with complete penetrance. Various phenotypes are described on the basis of site, appearance and progression of opacity in the lens. Both clinical and genetic heterogeneity of cataract have been reported [1,2]. The human lens is made up of elongated lens fiber cells, which are devoid of cell organelles. But the lens epithelial cells are actively dividing cells, which are nucleated and only during their differentiation and migration lose their DNA along with other cellular organelles [3]. New fiber cells are added from the periphery of the existing cells. The membrane of the lens fiber cells play an important role in the maintenance of homeostasis as it is involved in transport of water, ions, and energy for lens fiber cells. Generally, cataract is caused by alteration in spatial arrangement of proteins in the lens fibers. Crystallins α-, β- and γ- are the major protein components of the lens [4-6]. They undergo protein–protein interactions as core biophysical properties of crystallins to impart unique function to the eye [7]. The proteins once formed remain for the individual's lifetime since there is no major protein turnover in the lens. In addition mitochondrial debris is also reported to be present and may cause reduced vision [8].

Mutations in various genes have been reported to be associated with various phenotypes and they include crystallins, connexins, aquaporin, beaded structural filament protein 2, transcription factors like paired-like homeodomain 3 (*PITX3*) and heat shock transcription factor 4 (*HSF4*), avian musculoaponeurotic fibrosarcoma (*MAF*) protein and chromosome modifying proteins. Up to today, 39 loci have been mapped as candidate for cataractogenesis, of which 26 have been found to be associated with mutations in specific genes [9]. Majority of the mutations are reported to reside in genes coding for crystallins (50%), followed by connexins (25%) and the rest are distributed in various transcription factor (*HSF4*), and membrane proteins (beaded filament structural protein [*BFSP*] and lens intrinsic membrane protein 2 [*LIM2*]).

The human mitochondrial genome is circular, double-stranded DNA of 16,569 base pairs (bp) in length, located within the mitochondrial matrix and present in thousands of copies in every cell [10]. The electron transport chain, rRNA and tRNA are coded by mitochondrial genes. Mitochondrial DNA (mtDNA) is inherited through maternal lineage and is highly polymorphic. Currently used criteria for determining the likelihood that a missense mutation is pathogenic include: alteration in the evolutionarily conserved amino acid; absence of the mutation in controls; clinical features commonly linked to known pathogenic mtDNA mutations, defects in mitochondrial morphology, number and enzyme activities [11]. Pathogenic mtDNA mutations are typically characterized by incomplete penetrance, even when homoplasmic, presumably reflecting interactions with environmental and nuclear genetic factors [12]. As a result, inherited mtDNA mutations may not manifest the same pathogenicity in all the offspring of an affected mother [13,14]. Thus mtDNA mutations identified in rare families or subjects with a putative mitochondrial genetic disorder are often uncertain of its pathogenic significance. The mtDNA is highly polymorphic, and certain polymorphisms are thought to be risk factors in complex diseases such as diabetes mellitus [15], Alzheimer disease [16], Parkinson disease [17-20], bipolar disorder [21,22], and some types of cancer [23,24]. Over 100 mtDNA point mutations associated with human diseases have been identified, of which about 45 are missense mutations in protein encoding genes. However, there is no report up to today on complete mtDNA variations in childhood cataract. Hence, this study was aimed to examine the mtDNA mutation(s) in childhood cataract.

## Methods

### Subjects and methods

Seventeen families with childhood cataract, hailing from the coastal Karnataka region of India, who are attending the Kasturba Hospital (KH), Manipal, India and Government Wenlock Hospital (GWH), Mangalore, India, were recruited for this study. Clinical information of the probands was evaluated by a team of expert ophthalmologists and the details were recorded. Affected status was determined by a history of cataract extraction or ophthalmologic examination, which included slit lamp examination with dilated pupils, visual acuity testing, intraocular pressure measurement, and fundus examination by ophthalmologists. We studied the mtDNA mutations in three families with childhood cataract showing maternal inheritance. The patients with a history of trauma, intrauterine infection and metabolic disorders were excluded. Detailed pedigree of the kindred was ascertained by interviewing the parents or available family member(s). The clinical details of the patients who previously had cataract extraction were obtained through medical records. The inclusion criteria followed for selection of cataract families consisted of affected mother with affected as well as unaffected children available for the study. Four age and ethnically matched healthy individuals representing each family were included as control set. We have also earlier ruled out the presence of candidate gene mutations in these families [25]. We have included “syndromic cataract” as one of the exclusion criteria in our study and have considered only non-syndromic families.

### Sample collection

About 8–10 ml of peripheral blood samples were collected in EDTA vacutainer (BD, Biosciences, Franklin Lakes, NJ) from all participating individuals after obtaining their informed written consent in accordance with the Declaration of Helsinki and the Kasturba Hospital Institutional Ethics Committee. The blood samples were retained at 4 °C before processing.

### Isolation and amplification of DNA

Genomic DNA was extracted from peripheral lymphocytes, using standard phenol:chloroform method [26] with some modifications. To amplify the entire mtDNA, PCR was performed using previously defined 24 pairs of overlapping primers (BioServe, Hyderabad, India) [27] and presented in Table 1. Each PCR reaction (25 µl) was performed with 50 ng of genomic DNA, 1× PCR buffer, 1.5 M MgCl_2_, 200 µM dNTPs, 10 pmol each of sense and antisense primers and 1 U of Taq DNA polymerase (Finnzymes, Vantaa, Finland). PCR was performed in VERITI 96 well thermal cycler (Applied Biosystems, Foster City, CA) with an initial denaturation at 94 °C for 1 min followed by 35 cycles of denaturation at 94 °C for 30 s, primer annealing for 45 s at 61 °C and primer extension at 72 °C for 2 min. Following this a final extension was carried out at 72 °C for 5 min (Table 1) [27]. PCR products were purified using QIA quick purification kit (Qiagen, Hilden, Germany).

**Table 1:** Details of PCR primers used for mtDNA amplification [27].

**Name**	**Primer sequence 5’→3’**	**Product size**
MIT1F	CTCCTCAAAGCAATACACTG	840
MIT1R	TGCTAAATCCACCTTCGACC	
MIT2F	CGATCAACCTCACCACCTCT	802
MIT2R	TGGACAACCAGCTATCACCA	
MIT3F	GGACTAACCCCTATACCTTCTGC	860
MIT3R	GGCAGGTCAATTTCACTGGT	
MIT4F	AAATCTTACCCCGCCTGTTT	887
MIT4R	AGGAATGCCATTGCGATTAG	
MIT5F	TACTTCACAAAGCGCCTTCC	832
MIT5R	ATGAAGAATAGGGCGAAGGG	
MIT6F	TGGCTCCTTTAACCTCTCCA	898
MIT6R	AAGGATTATGGATGCGGTTG	
MIT7F	ACTAATTAATCCCCTGGCCC	975
MIT7R	CCTGGGGTGGGTTTTGTATG	
MIT8F	CTAACCGGCTTTTTGCCC	814
MIT8R	ACCTAGAAGGTTGCCTGGCT	
MIT9F	GAGGCCTAACCCCTGTCTTT	827
MIT9R	ATTCCGAAGCCTGGTAGGAT	
MIT10F	CTCTTCGTCTGATCCGTCCT	886
MIT10R	AGCGAAGGCTTCTCAAATCA	
MIT11F	ACGCCAAAATCCATTTCACT	987
MIT11R	CGGGAATTGCATCTGTTTTT	
MIT12F	ACGAGTACACCGACTACGGC	900
MIT12R	TGGGTGGTTGGTGTAAATGA	
MIT13F	TTTCCCCCTCTATTGATCCC	816
MIT13R	GTGGCCTTGGTATGTGCTTT	
MIT14F	CCCACCAATCACATGCCTAT	940
MIT14R	TGTAGCCGTTGAGTTGTGGT	
MIT15F	TCTCCATCTATTGATGAGGGTCT	891
MIT15R	AATTAGGCTGTGGGTGGTTG	
MIT16F	GCCATACTAGTCTTTGCCGC	840
MIT16R	TTGAGAATGAGTGTGAGGCG	
MIT17F	TCACTCTCACTGCCCAAGAA	802
MIT17R	GGAGAATGGGGGATAGGTGT	
MIT18F	TATCACTCTCCTACTTACAG	866
MIT18R	AGAAGGATATAATTCCTACG	
MIT19F	AAACAACCCAGCTCTCCCTAA	977
MIT19R	TCGATGATGTGGTCTTTGGA	
MIT20F	ACATCTGTACCCACGCCTTC	970
MIT20R	AGAGGGGTCAGGGTTGATTC	
MIT21F	GCATAATTAAACTTTACTTC	938
MIT21R	AGAATATTGAGGCGCCATTG	
MIT22F	TGAAACTTCGGCTCACTCCT	1162
MIT22R	AGCTTTGGGTGCTAATGGTG	
MIT23F	TCATTGGACAAGTAGCATCC	765
MIT23R	GAGTGGTTAATAGGGTGATAG	
MIT24F	CACCATCCTCCGTGAAATCA	954
MIT24R	AGGCTAAGCGTTTTGAGCTG	

The annealing temperature for all primers were found to be 61 °C.

### Sequencing and analysis of complete mitochondrial genome

The purified amplicons were sequenced using the ABI Big Dye Terminator cycle sequencing kit, (Applied Biosystems). Cycling conditions used were: 25 cycles of denaturation at 96 °C for 10 s, annealing at 50 °C for 5 s, and extension at 60 °C for 4 min. Extended products were purified and analyzed using ABI 3130 Genetic Analyzer (Applied Biosystems). Sequences obtained were aligned with the revised Cambridge reference sequence (rCRS) [28] and the mutations were recorded. The observed alterations were compared with mitochondrial databases such as Mitomap and mtDB as well as with Cat-Map database for their significance. The mitochondrial sequences variations which were found in the affected individuals while absent in unaffected members of the same family were referred to as unique.

## Results

### Clinical details

**Figure 1:**
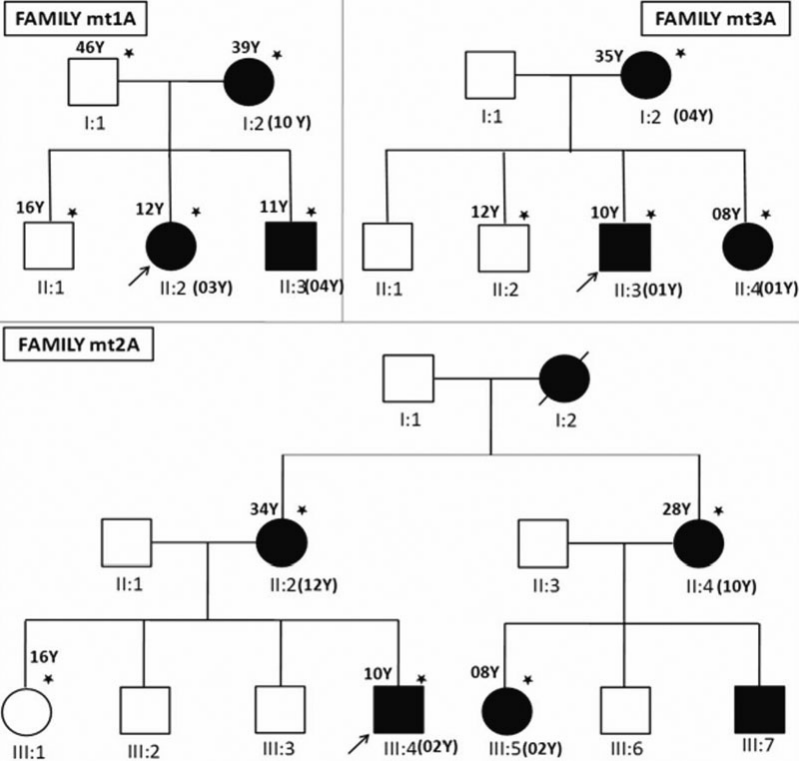
Pedigrees of the families with inherited cataract included in the study. All the families showed maternal inheritance. The dark squares and circles represent affected male and female, respectively. The arrow indicates the proband and asterisk indicates the members involved in the study. Both the sexes were affected. Y represents the age in years. The age at onset is represented in bracket next to the affected member.

**Figure 2:**
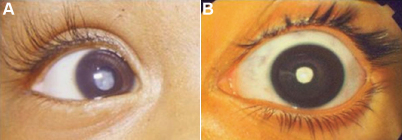
Cataract phenotypes. The eye images presenting cataract phenotypes in proband of childhood cataract family mt2A (III:4; **A**) and family mt3A (II:3; **B**) showing white central dense opacity leading to total cataract. The progression of cataract was observed to be differential in each eye. The zonular cataract phenotype of family mt1A proband (II:2) is not shown.

All the participants were free from other diseases such as congenital rubella and vitamin A deficiency and showed maternal inheritance of cataract (Figure 1 and Table 2). The degree of opacity of the eyes varied greatly showing different types of morphology as identified by slit lamp or by direct flash light examination. The probands of family mt2A (III:4) and mt3A (II:3) showed more severe phenotype with central white opacity leading to total cataract (Figure 2A,B). The proband of family mt3A (II:2) showed bilateral zonular cataract and we do not have the image of the cataract phenotype for same. In all the cases, the disease was found to be progressive in nature with the mean age of 5 years. The varied degree of opacity among the family members is suggestive of the presence of probable modulators playing a critical role in disease manifestation.

**Table 2:** List of variations identified in healthy and cataract samples in relation to reference sequence (NC_012920).

**Locus**	**Nucleotide change**	**Amino acid change**
*12S rRNA*	A1438G	——–
*16S rRNA*	A1811G, A2706G, T2746C, Del3106C	——–
*ND1*	T3394C, A3397G, A3434G, G3483A, A3511G G3693A, T3847C, C3921A, T4023G, C4171T	Y30H, M31V, Y43C, E59E, T69A, L129L, L181L, S205S, T239T, L289L
*T1*	T4291C	——–
*CO1*	**C6452T**, **Ins6524A/T**, C7028T, **T7302C**, **T7319C**	**L183L**, **FmsftT207**, A375A, **L467L**, **I472I**
*ATP6*	**A8679G**, A8701G, T8843C, A8860G, **A9135G**	**K51K**, T59A, I106T, T112A, **E203E**
*CO3*	T9540C, **A9632C**	L112L, **V142V**
*TG*	T10031C	——–
*ND3*	A10398T, C10400T	T114S, T114T
*ND4L*	G10685A	A72A
*ND4*	**C10797T**, T10873C, A11467G, G11719A, G12007A	**P13L**, P38P, L236L, G320G, W416W
*tRNA*	A12308G	——–
*ND5*	G12372A, **A12999G**, **C13668T**, **A13956C**, **C14109T**	L12L, **A221A**, **N444N**, **L540L**, **F591F**
*ND6*	G12501A	M55M
*ND5*	T12696C, C12705T, A12999G, C13668T, C13943T, A13956C, C14109T	Y120Y, I123I, A221A, N444N, T536M, L540L, F591F
*ND6*	**C14155T**	**G173G**
*Cytb*	C14766T, T14783C, G14905A, G15043A, T15155C, A15133G, G15301A, A15326G	T7I, L13L, M53M, G99G, T123T, M129M, L185L, T194A
*D-loop*	A93G, A153G, T152C, A73G, G66T, Ins65T, Del58T, Del57T, Ins55T, A16206C, C16223T, T16311C, **T16445C**, T16519C, C16527T	——–

The novel mutations observed in inherited cataract families are highlighted in bold.

### Sequence analysis of mtDNA

**Figure 3:**
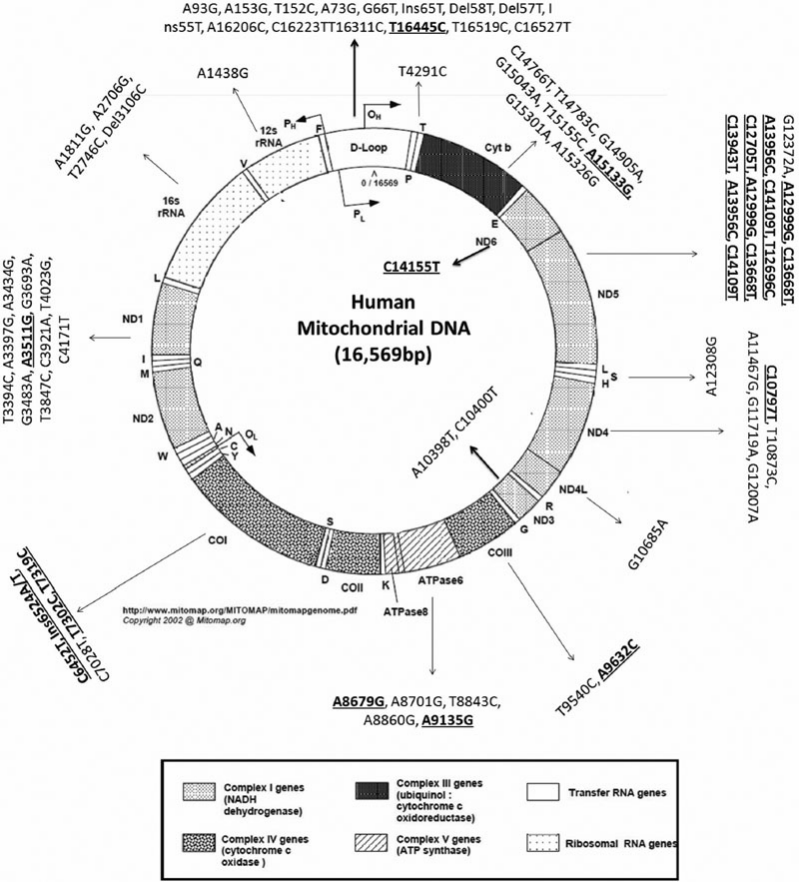
The distribution of mitochondrial sequence variations identified. The sequence variations identified in the coding and non-coding regions of the mitochondria are shown by arrow heads. The D- loop showed highest number of variation followed by ND1 region and Cytb. The novel mutations observed in inherited cataract families are highlighted in bold and underlined. The circular mitochondrial genome map was retrieved from mitomap.

We have sequenced the complete mtDNA of 14 individuals, with 10 childhood cataract patients belonging to 3 cataract families and 4 healthy individuals. A total of 72 nucleotide variations were observed, of which 15 were in the major non-coding D-loop region, 3 in the tRNA genes, 5 in the rRNA genes, and 49 in the protein-coding region (Figure 3). Of the total 72 variations, 56 were reported previously and 16 were novel, of these one was in the non-coding region, 12 were synonymous substitutions, 2 were non-synonymous substitutions and one was frameshift mutation (Figure 3).

**Figure 4:**
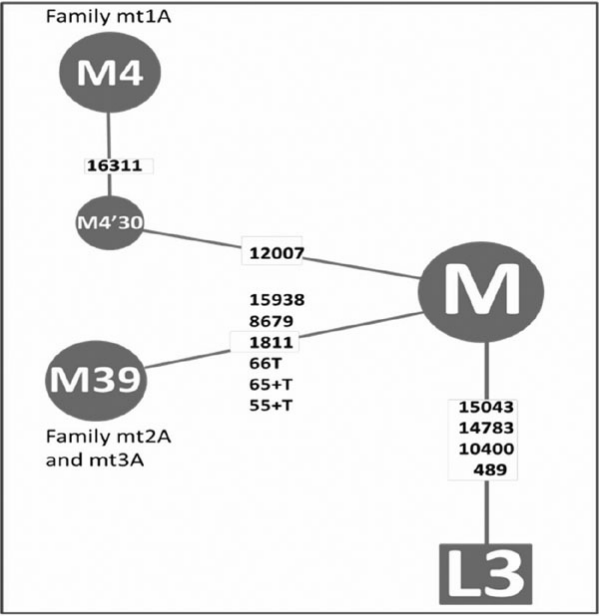
Mitochondrial haplogroup analysis between childhood cataract families. The map generated for the mitochondrial alterations for each family was analyzed for haplogroups. Family mt1A showed M4 haplogroup and family mt2A, mt3A fall in to M39 haplogroups. The phylogenetic networks and evolutionary branching was constructed using Network 4.5.1.0. The reduced median or RM network algorithm was used providing binary data of the mitochondrial genomic variations. M-Macrohaplogroup, M4’30- Superhaplogroup, M4 haplogroup for family mt1A and M39 haplogroup for families mt2A, mt3A.

The map generated for the mitochondrial alterations for each family was analyzed for haplogroups. Family mt1A showed M4 haplogroup and family mt2A, mt3A fall in to M39 haplogroups (Figure 4).

The phylogenetic networks and evolutionary branching was constructed using Network 4.5.1.0. The reduced median or RM network algorithm was used providing binary data of the mitochondrial genomic variations.

The SNPs at position 1438, 2706, 3106, 7028, 8860, and 15326 were found in all the samples. These variations were seen in very high frequency in this population. Of these 8860 and 15326 had a Grantham value of 58 and are reported in subfertile, abdominal aortic aneurysm, AD, PD, T2DM w/wo angiopathy, LHON, and dystonia [13,15,16,19,20,29-31]. Among the other SNPs with high Grantham value include 3394 and 13943, found in over 80 cataract samples. The SNPs at 8701 and 10398 showed a Grantham value of 58 but were observed in both cataract and healthy samples. The SNP 3434 showed highest Grantham value of 194 but was found to be in only healthy subjects.

A3511G leading to Thr69Ala was observed in the mt2A cataract family. Four variations were found in the cytochrome oxidase subunit I (*COI*) region at nucleotide positions 6452, 6524, 7302, and 7319, of these substitutions, those at 6524, 7302, and 7319 were synonymous. The variation at position 6452 was seen in three individuals of family mt3A; of which one was a healthy individual and two were cataract patients. Two synonymous variations namely A8679G and A9135G were observed in the ATP6 region. Of these, A8679G was found in 3 members of the family, mt3A, and among these three one was in healthy individual and other two were observed in patients with cataract. A9135G was observed in two cataract patients of the family mt1A and in all the members of the family mt2A; of which one was healthy and the other four were in affected individuals. C10797T leads to Pro13Leu and was observed in three affected members of the family mt1A in the nicotinamide adenine dinucleotide dehydrogenase subunit 4 (*ND4*)region.

Among the sixteen novel mutations; four were observed in the *ND5* gene and all four were synonymous. A12999G was observed in one healthy person and three in affected individuals of family mt3A; C13668T was observed in two affected members of the family mt2A; A13956C was seen in one healthy individual of the family mt1A; C14109T was observed in two and three individuals of families mt1A and mt2A, respectively. C14155T synonymous mutation in the ND6 gene was observed in only one affected member of the family mt3A. A15133G was observed in the ubiquinol-cytochrome-c reductase complex cytochrome b subunit (MT-*CYB*) gene of a healthy individual of the family mt1A. Among the 16 novel variations only one was observed in the D-loop region (16445) of all members of the family mt3A.

Among the 72 nucleotide variations observed in the families; mt1A, mt2A and mt3A; four (T4023C, T4291C, C10797T, and T15115C) were unique to the affected individuals of mt1A family and seven (T3394C, A3511G, C4171T, G12372A, G12501A, T12696A, and C13943T) were unique to the mt2A family and one variation (A3511G) was unique to mt3A family (Table 3). Among the four nucleotide substitutions observed in the mt1A family, two were synonymous (NADH dehydrogenase subunit-1: T239T, CYB-T123T), one was non-coding, and one was a non-synonymous (ND4-P13L). T4023C mutation in the NADH dehydrogenase subunit-1 gene was observed in three affected members and a non-synonymous C10797T (P13L) was observed in two affected individuals (I:2, II:2). T15115C in the CYB region was observed in all three affected members of this family (mt1A).

**Table 3:** List of variations unique to inherited cataract samples.

**Family**	**Location**	**Nucleotide position**	**Amino acid position**
Family mt1A	MT-ND1	T 4023 C	T 239 T
	MT-T1	T 4291 C	Non coding
	MT-ND4	C 10797 T	P 13 L
	MT-CYB	T 15115 C	T 123 T
Family mt2A	MT-ND1	T 3394 C	Y 30 H
	MT-ND1	A 3511 G	T 69 A
	MT-ND1	C 4171 T	L 289 L
	MT-ND5	G 12372 A	L 12 L
	MT-ND5	G 12501 A	M 55 M
	MT-ND5	T 12696 C	Y 120 Y
	MT-ND5	C 13943 T	T 536 M
Family mt3A	MT-ND1	A 3511 G	T 69 A

The term ‘unique’ refers to mitochondrial sequence alterations found in inherited cataract samples while absent in unaffected members of the same family.

**Figure 5:**
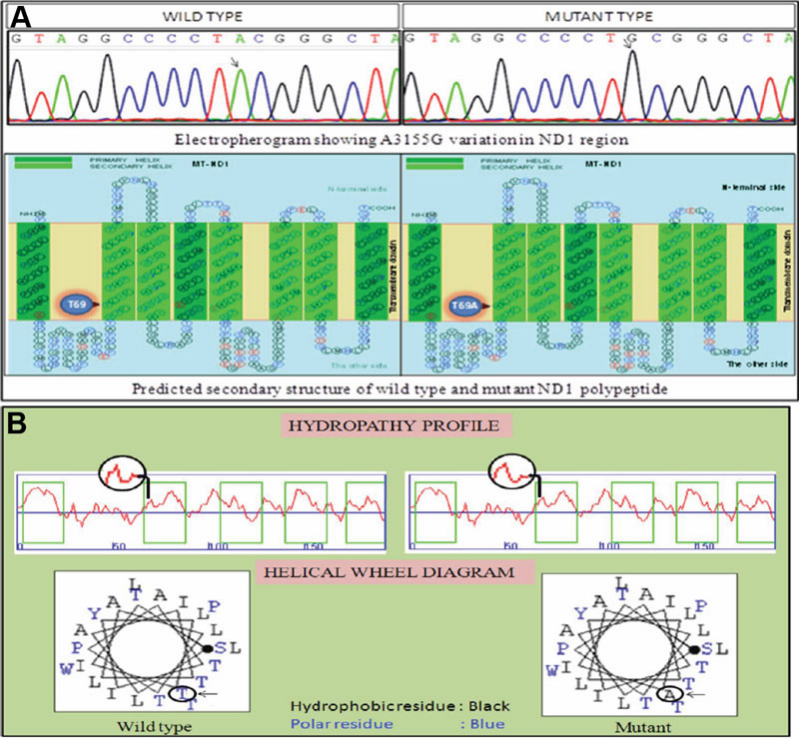
Electropherogram and polypeptide of wild type and mutant sequence of ND1 region. **A**: Electropherogram showing wild type and mutant nucleotide sequence of ND1 region with A>G transition at 3155 nucleotide position. Predicted secondary structure of the wild type and mutant ND1 polypeptide showing the altered amino acid. **B**: The architecture of the membrane proteins is reflected in the hydropathy profile. The helical wheel diagram of predicted polypeptide show change from polar residue to hydrophobic residue. Arrow mark indicates the altered aminoacid position. The source of the structure prediction is SOOSUI.

Among the seven mutations which were unique to the second family (mt2A), four were synonymous and three were non-synonymous. In the ND1 region, three substitutions were observed in four affected members and of these T3394C (Y30H) and A3511G (T69A) were non-synonymous and C4171T (L289L) was synonymous substitution. Four nucleotide variations unique to the affected individuals were in the ND5 gene and of these, three were synonymous mutations (T12696C, G12372A, and G12501A) and one was non-synonymous C13943T (T536M) observed in four members of the second family mt2A. The A3511G mutation (T69A) in the ND1 region was the only common mutation in affected members of mt3A family. All these findings show that T69A is the common mutation in affected members in two different childhood cataract families mt2A and mt3A (Table 3). The secondary structure of the wild type and T69A mutant ND1 polypeptide showed changes in the transmembrane domain of mutant polypeptide. The source of the structure prediction is shown (Figure 5).

## Discussion

In recent years, somatic mitochondrial mutations have been identified and implicated in the pathogenesis of many diseases [32-36]. Further studies have reported identification of mitochondrial mutations in various eye disorders including cataract and congenital cataract [32,33,37,38]. In the present study, we perhaps for the first time sequenced the entire mitochondrial genome in patients having congenital cataract to identify the role of mtDNA mutations if any in childhood cataract. In our patients, the variation affected ten protein coding genes (ND1, CO1, ATP6, CO3, ND3, ND4L, ND4, ND5, ND6, ND5, ND6, and Cytb), two rRNA genes (12s rRNA and 16srRNA) and non-coding region namely Displacement loop (D-Loop). In our study we have identified 72 nucleotide variations in mitochondria, of which 15 were observed in the major non-coding D-loop region, 3 in the tRNA genes, 5 in the rRNA genes and 49 in the protein coding region. Among the variations 56 were reported previously and 16 were novel of which one was in the non-coding region, 12 synonymous substitutions and 2 were observed to be non-synonymous substitutions along with a frame shift mutation. Further, the D-loop was found to be the hot spot for mitochondrial variations. Among the variations identified 65.05% was observed from coding region indicating the strong involvement of mitochondrial variation and oxidative damage in the pathogenesis of congenital cataract. In the coding region, the ND1 region (10 variations) was the most frequently altered one followed by Cytochrome-b (Cytb) (8 variations). Taken together, our sequencing results point out that the high incidence of mtDNA variations might lead to alteration in aerobic respiration, replication and transcription of mitochondria during development of eye and this might play essential role in congenital cataract.

We have performed the clinical, genetic and molecular characterization of hereditary cataract families. The visual loss as a sole clinical phenotype was only present in the maternal lineage of these pedigrees, suggesting the involvement of mtDNA mutation(s) in the pathogenesis of childhood cataract. Moreover, these individuals were excluded for the involvement of *CRYAA, CRYBB2, CRYGA, CRYGB, CRYGC, CRYGD, GJA3, GJA8*, and *PAX6* [25] gene mutations which are responsible for approximately 90% of the cases. Sequencing of the complete mitochondrial genome led to the identification of several homoplasmic mutations, specific to individuals with cataract. Clinical and genetic evaluations revealed the variable severity and age-at-onset in visual loss. Notably, there was a typical difference in vision loss and phenotypes in all the three pedigrees. In contrast to the mitochondrial variations reported in other disorders such as specific late-onset neurodegenerative diseases [30], cancer [39], and Noonan syndrome [40], the variations identified in these families do not share the similarity. This observation is in agreement with the phenotypic heterogeneity among the family members. Since we observed several novel and reported mutations in individuals with cataract, we presume that the mitochondrial damage could be more in affected individuals compared to healthy individuals. This would lead to accumulation of mtDNA damage and ROS generations finally leading to the damage of lens proteins.

The whole genome screening for mutations/SNPs not only provided the information on the sequence variations in childhood cataract but also helped in comparing the mutations with the previous reports for other diseases. To the best of our knowledge, this is the first report on mtDNA sequence variation among the cataract patients of Indian sub-continent. This study indeed may serve as reference for those who would work on cataract in future. The mtDNA mutations, which are present only in affected but not in healthy individual of the family, is of interest and indicate the possible role of these mutations with disease manifestation.

Mutations at the nucleotide positions 8701 and 10398 have intracellular functions such as calcium-signaling or pH regulation [41]. Mitochondrial function is multifaceted, and various mitochondrial abnormalities are observed to be variable and currently unpredictable effects on different tissues. Our study evaluated the mitochondrial sequence of childhood cataract individuals and addressed the impact of mitochondrial mutations/polymorphisms on biologic perspectives of the lens. This evaluation may not be comprehensive, but these mitochondrial variations provide a base line and also help to correlate the findings with reported variation in other pathological conditions. The localization, mitotic segregation coupled with tissue specific expression of such mutated mitochondria have already been reported in mitochondrial diseases. Additionally, that the growing evidences in mitochondrial studies in cataract have suggested the mtDNA sequence variation and its association with cataract show variation with disease phenotype. None of the studies was able to provide the mechanism as to how such sequence variations will promote cataractogenesis. Thus further work on functional studies needs to be undertaken to delineate the molecular mechanism behind mitochondrial sequence variations in congenital cataract.

Mitochondrial DNA variation is reported in cataract and other eye related disorders previously including congenital cataract [32,33,42,43]. However, none of the studies has sequenced the entire mtDNA in congenital cataracts. The present study highlights the importance of defining the precise nature of mutation which can be used for patient diagnosis as well as for genetic counseling of maternal lineage relatives. ND gene of mtDNA being highly altered in general and found to contain 4 variations specific for cataract patients of the first family, six for the second and one for the third family. An earlier study has reported the congenital cataract as first symptom of a neuromuscular disease caused by a novel single large-scale mitochondrial DNA deletion [32]. Some of the sequence variations found in the mtDNA are found to be associated with cataract and or with other diseases with cataract as one of the symptoms [32,44-46]. Additionally some of them such as T152C, A73G, C16223T, T16311C, G11778A, T3394C, and C14766T were reported to be associated with LHON and other ocular disorders.

The A1438G mutation in the 12S rRNA gene has been described in Japanese type 2 diabetic group as well as in some patients with Parkinson disease [47,48]. The T3394C and G11719A variation of *ND1* and *ND4* genes is reported in LHON [49,50]. The variant C7028T in the *MT-CO1* gene was reported in ovarian cancer patients and Parkinson disease [51,52]. The variant A8679G in *ATPAse6* was reported in patients having Persian LQTS [53]. The A8701G variant detected in our study was previously identified as altering mitochondrial matrix pH and intracellular calcium dynamics and was suspected to be involved in pathogenesis of diseases [41]. Further, we have also identified variants which are reported in schizophrenia, bipolar disorder, and major depressive disorder [54]. The A12308G variation in tRNA gene detected in our study is also previously reported in congenital glaucoma [55] and was shown to be associated with increased ROS production. One can thus assume that the increased ROS production due to mtDNA alteration during the development of eye might play significant role in cataractogenesis [56].

The T3394C, which lead to Y30H in MT-ND1, was observed in 40% of the study subjects, whereas it was observed in less than 4.5% of other studies [40,57-60]. The T4023C silent mutation was observed at a frequency of 30% in the present study, whereas in other studies, it was reported to be only at 2% [57,59-61]. The C4171T in MT-ND1 which lead to a synonymous effect at the 289 position was observed in 30% of our study population, and it was reported in less than 1.5% in other studies [62]. The T12696C in MT-ND5, a silent mutation was observed in 40% of the individuals studied; whereas, in other studies [57,63] it was reported to be less than 4.5%. The C13943T transition in MT-ND5, which led to T536M, was observed in 40% of the individuals in the present study, whereas in another study it was reported to be less than 0.5% [57]. The T15115C substitution in MT-CYB was observed in 30% in the present study, compared to less than 5.5% reported earlier [57]. The mitochondrial DNA haplogroup associations are shown in Noonan syndrome and this opens up new approaches to understand disease associations with specific haplogroups [64].

Our study suggests that there is an involvement of mitochondrial genome variation with childhood cataract even though the human lens fiber cells lack mitochondria. The novel mutation T69A was found to be common in affected members of two families (mt2A, mt3A), and P13L mutation was seen in affected individuals of one childhood cataract family (mt1A). By observing a common haplogroup 'M39' in two cataract families mt2A and mt3A we do not claim its proximity to disease susceptibility. It could be due to the same ethnic background of both families since this haplogroup is even reported in other sub populations across southern India. From our observations, it is not possible to generalize association of any haplogroup with susceptibility to inherited cataract. More research in this area may advance toward developing an interactive relationship between nuclear genes and mitochondrial DNA in protecting the lens proteins.

Taken together, our analysis show that mtDNA variations are commonly found in congenital cataract, but the precise role of such alteration is not clear. Further, additional functional studies needs to be undertaken to understand how these individual variants alone or in combination bring about cataract phenotypes in those patient who harbor such mtDNA variations. Sligh et al. [65] showed that maternal germ line transmission of mutant mtDNAs in chimeric mouse model exhibited ocular abnormalities including congenital cataracts and functional retinopathy. Currently it is unknown as to how mitochondrial variations lead to congenital cataract, however it may be speculated as lens in its early stages of development may be subjected to impaired homeostasis through ROS coupled energy crisis from defective mtDNA. Further, this in turn might alter lens fiber as well as some of the crystalline and associated proteins during early eye development leading to cataractogenesis [65].

The results presented here suggest that the large number of variations in the mitochondrial genome in the childhood cataract patients thus, may act in synergy with the nuclear variations of either candidate genes or other unknown genes and this remains to be elucidated. Data from such a study may help in understanding the mechanism of cataractogenesis.
